# Lipidomics profiling reveals distinct patterns of plasma sphingolipid alterations in Alzheimer’s disease and vascular dementia

**DOI:** 10.1186/s13195-023-01359-7

**Published:** 2023-12-12

**Authors:** Xin Ying Chua, Federico Torta, Joyce R. Chong, Narayanaswamy Venketasubramanian, Saima Hilal, Markus R. Wenk, Christopher P. Chen, Thiruma V. Arumugam, Deron R. Herr, Mitchell K. P. Lai

**Affiliations:** 1 Department of Pharmacology, Yong Loo Lin School of Medicine, National University of Singapore, Singapore, 117597 Singapore; 2 Singapore Lipidomics Incubator (SLING), Department of Biochemistry, Yong Loo Lin School of Medicine, National University of Singapore, Singapore, Singapore; 3 Memory, Aging and Cognition Centre, National University Health System, Singapore, Singapore; 4 Raffles Neuroscience Centre, Raffles Hospital, Singapore, Singapore; 5 Saw Swee Hock School of Public Health, National University of Singapore, Singapore, Singapore; 6 Centre for Cardiovascular Biology and Disease Research, Department of Microbiology, Anatomy, Physiology and Pharmacology, School of Agriculture, Biomedicine and Environment, La Trobe University, Melbourne, VIC Australia; 7 Center for Genetic Disorders and Aging Research, Sanford Burnham Prebys Medical Discovery Institute, La Jolla, CA USA

**Keywords:** Alzheimer’s disease, Biomarkers, Lipidomics, Sphingolipids, Vascular dementia

## Abstract

**Background:**

Alzheimer’s disease (AD) and vascular dementia (VaD) are two of the commonest causes of dementia in the elderly. Of the myriad biomolecules implicated in dementia pathogenesis, sphingolipids have attracted relatively scant research attention despite their known involvement in multiple pathophysiological processes. The potential utility of peripheral sphingolipids as biomarkers in dementia cohorts with high concomitance of cerebrovascular diseases is also unclear.

**Methods:**

Using a lipidomics platform, we performed a case–control study of plasma sphingolipids in a prospectively assessed cohort of 526 participants (non-cognitively impaired, NCI = 93, cognitively impaired = 217, AD = 166, VaD = 50) using a lipidomics platform.

**Results:**

Distinct patterns of sphingolipid alterations were found in AD and VaD, namely an upregulation of d18:1 species in AD compared to downregulation of d16:1 species in VaD. In particular, GM3 d18:1/16:0 and GM3 d18:1/24:1 showed the strongest positive associations with AD. Furthermore, evaluation of sphingolipids panels showed specific combinations with higher sensitivity and specificity for classification of AD (Cer d16:1/24:0. Cer d18:1/16:0, GM3 d16:1/22:0, GM3 d18:1/16:0, SM d16:1/22:0, HexCer d18:1/18:0) and VAD (Cer d16:1/24:0, Cer d18:1/16:0, Hex2Cer d16:1/16:0, HexCer d18:1/18:0, SM d16:1/16:0, SM d16:1/20:0, SM d18:2/22:0) compared to NCI.

**Conclusions:**

AD and VaD are associated with distinct changes of plasma sphingolipids, warranting further studies into underlying pathophysiological mechanisms and assessments of their potential utility as dementia biomarkers and therapeutic targets.

**Supplementary Information:**

The online version contains supplementary material available at 10.1186/s13195-023-01359-7.

## Introduction

Dementia is a progressively debilitating disease, afflicting an estimated 47.5 million people worldwide and up to 10% of community dwelling older adults in Singapore [1, 2]. The high prevalence of dementia has many social and economic implications as well as burden on the healthcare system and society, highlighting the importance of dementia research and prevention. The most common cause of dementia is Alzheimer’s Disease (AD), characterised by neurodegeneration associated with abnormally aggregated β-amyloid and tau proteins. However, there are multiple other causes of dementias, with varying and often overlapping pathologies including amyloidosis, neurodegeneration, cerebrovascular disease and inflammation [3–6]. Apart from AD, Vascular dementia (VaD) is the second most common cause of dementia [7]. It is characterised by the presence of cerebrovascular disease such as infarcts, microbleeds, cerebral amyloid angiopathy [8, 9]. These features are not only observed in VaD patients, but also in patients with AD [10].

**Fig. 1:**
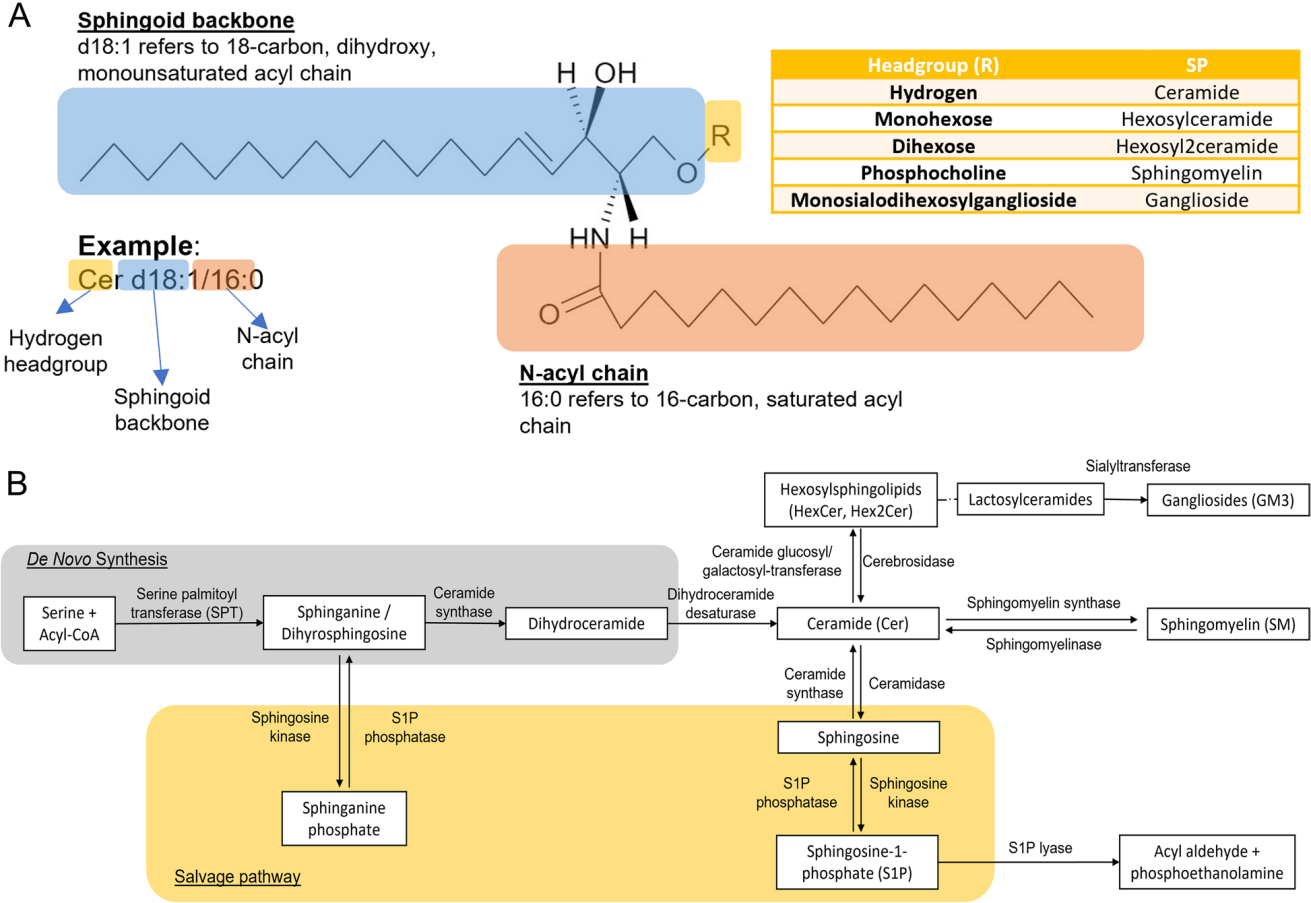
**A** Structural components and naming convention of sphingolipids (SP). The three major moieties in SPs are indicated, with sphingoid backbone highlighted in blue, n-acyl chain attached to backbone via amide bond in orange, and the headgroup in yellow. Headgroups of SPs measured in this study are summarized in the inset table. SPs in this manuscript were named by the class abbreviation (SM, Cer etc.), followed by the sphingoid backbone, then followed by the n-acyl chain (both indicated as number of carbons and number of double bonds in the acyl chain). **B** Depiction of the sphingolipid metabolic pathway, including the de novo synthesis pathway highlighted in grey and the salvage pathway highlighted in yellow. Boxes indicate the sphingolipids or their substrates. Enzymes catalysing the reactions are indicated above the arrows. Dotted line represents a subset of the sphingolipids, in this case, lactosylceramide is a dihexosylceramide

Sphingolipids have physiological and pathophysiological functions in cellular processes. Recent research has investigated their alterations in disease, as well as their functions within the nervous system [11–13]. Their alterations have been suggested to play a role in dementia pathology and pathogenesis including, for example, accelerating processes such as amyloid production and apoptosis [13, 14]. Research in this field has also gained traction in recent years, especially with the advent of highly sensitive lipidomics platforms [13, 15]. Sphingolipids consist of multiple species including sphingomyelins, ceramides and sphingosines which can be interconverted by a well-characterized metabolic pathway (Fig. 1). The levels of each species may be affected by one another and thus, it is imperative to look at all species of sphingolipids in relation to each other.

## Materials and methods

### Study cohort, medical and cognitive assessments

The selection and assessment of the cohort for this case–control study, which represents baseline measurements of an ongoing longitudinal study, have been previously described [16, 17] (also see Supplementary Table 1). Briefly, patients with subjective complaints of memory loss were recruited from memory clinics at Singapore’s National University Hospital and St Luke’s Hospital sites. All subjects underwent clinical, physical, neuropsychological assessments and neuroimaging at the National University of Singapore. Relevant demographic and medical information, including vascular risk factors and exclusion factors such as previous head trauma, thyroid disease, non-AD neurodegenerative conditions (e.g., Parkinson’s disease), and psychiatric illnesses, were collected by administering a detailed questionnaire and reviewing of medical records. Furthermore, subjects were administered a comprehensive neuropsychological test battery consisting of several domains, namely, executive function, attention, language, visuomotor speed, visuoconstruction, verbal memory and visual memory, along with standard cognitive assessments (Mini-Mental State Examination [18] and Montreal Cognitive Assessment [19], see Supplementary Table 2). Diagnoses of cognitive impairment and dementia were made at regular consensus meetings of study clinicians and neuropsychologists, where cognitive impairment, no dementia (CIND) cases were defined by people who did not meet the Diagnostic and Statistical Manual Fourth Edition (DSM-IV) diagnostic criteria for dementia [20] but showed impairment in one or more domains of the neuropsychological battery, as defined by education-adjusted scores ≥ 1.5 standard deviations below normal established means for at least half of the tests for that domain. AD cases were diagnosed using the National Institute of Neurological and Communicative Disorders and Stroke and the Alzheimer’s disease and Related Disorders Association (NINCDS-ADRDA) criteria [21], while vascular dementia (VaD) was diagnosed using the National Institute of Neurological Disorders and Stroke-Association Internationale pour la Recherché et l’ Enseignement en Neuroscience (NINDS-AIREN) criteria [22]. Non-cognitively impaired (NCI) controls were defined as those with subjective memory complaints, but who were found to be cognitively normal after undergoing objective neuropsychological assessments.

### Covariates

In addition to demographic information, medical histories of vascular risk factors such as hypertension, hyperlipidemia, diabetes, smoking and cardiovascular disease were collected and classified as absent or present. Hypertension was defined as systolic blood pressure ≥ 140 mmHg and /or diastolic blood pressure ≥ 90 mmHg or a history of hypertension, or use of antihypertensive medication. Hyperlipidemia was defined as total cholesterol level ≥ 4.14 mmol/l or a history of hyperlipidemia, or use of lipid-lowering medication. Diabetes mellitus was defined as glycated hemoglobin ≥ 6.5% or a history of diabetes mellitus, or the use of any glucose-lowering medication. Cardiovascular disease was determined by previous history of atrial fibrillation, congestive heart failure and / or myocardial infarction. Apolipoprotein E (APOE) genotyping were as previously described [23] for the determination of APOE ε4 carrier status, defined by the presence of at least one APOE ε4 allele.

### Blood processing

Non-fasting blood was collected via venipuncture from study participants into both serum-separating tubes (SST) and ethylenediaminetetraacetic acid (EDTA)-containing tubes, followed by centrifugation at 2000 g for 10 min at 4 °C.

### Liquid chromatography–tandem mass spectrometry (LC–MS/MS)

Lipids from plasma samples were extracted using a 1-butanol:methanol (1:1, v/v, Merck Millipore) extraction solvent containing a set of internal standards based on a method previously described [24]. Briefly, 100 μl of extraction solvent containing internal standards was added to 15 μl of each plasma sample, sonicated for 30 min, centrifuged at 16,000 g and 90 μl of lipid extract were then transferred into glass vials and stored at -80◦ C until analysis. The extracted lipids were analysed by positive mode electrospray ionization mass spectrometry using an Agilent 6495 QQQ mass spectrometer. Lipid separations were performed on a 1290 Infinity II ultra-high performance liquid chromatography system, using a reversed-phase Agilent ZORBAX Rapid Resolution High Definition Eclipse Plus C18 column. Lipids were quantified using a dynamic, multiple reaction monitoring method with measurement of peak area of quantifier transitions by peak integration. Lipid peaks were identified based on their specific precursor and product ion transitions in addition to their retention time [24]. Subsequent normalization with internal standards was carried out as previously discussed [24]. To ensure the quality and precision of the results, pooled quality control (QC) samples were included every 10 study samples. The coefficient of variation (CoV) of each individual lipid in the QC samples was then calculated, and lipids with CoV higher than 30% were excluded. Evaluation was performed for 177 peaks for each plasma sample. Of these, 77 peaks met our inclusion criteria for reliability and were included in subsequent analyses. These included members of the following classes: ceramides, cerebrosides (monohexosylceramides or “HexCer”), globosides (dihexosylceramides or “Hex2Cer”), gangliosides (GM3) and sphingomyelins (SMs) (Supplementary Table 3). Principal components analysis was performed, and principal components were plotted to identify any potential drifts or batch effects (Supplementary Figure 1).

### Statistical analyses

Statistical analyses were performed using Stata Version 14. Kruskal–Wallis analyses of variance (ANOVA) with post-hoc Dunn’s tests and Chi-square tests were used to compare the characteristics of the cases and control groups. Plasma SPs were compared between cases and controls using Mann–Whitney U test with adjustment for false discovery rate (FDR) using Benjamini-Hochberg (BH) method. Binary logistic regressions were conducted to evaluate association between plasma SPs and risk of CIND, AD and VaD, using log-transformed plasma SPs as independent variable. Unadjusted models were reported, as well as models adjusted for age, sex, education, and/or ApoE ε4 carrier status, and/or hypertension, diabetes, hyperlipidemia and cardiovascular disease. *P*-values were adjusted for FDR using BH method. Regression with Least Absolute Shrinkage and Selection Operator (LASSO) regularisation was performed on SPs that were significantly associated with AD in univariate analysis [25]. Five-fold cross-validation was used to identify the λ_1SE_ [26]. These were performed using lassopack in Stata Version 14 [27]. Receiver-operating characteristics (ROC) analyses were performed for baseline model and combined models to determine Area under curve (AUC) and likelihood ratio tests were performed to determine if models were significantly improved. Backward stepwise logistic regression was used by setting the significance level for removal at 0.1. For all analyses, *P*-values < 0.05 were considered statistically significant.

## Results

### Baseline characteristics of study cohort

A total of 526 participants were included in this study. Table 1 shows the main demographic and clinical characteristics of each cognitive subgroup. Participants with cognitive impairment were older, and a lower proportion had above primary education. A higher proportion also had history of hypertension, hyperlipidemia, diabetes and cardiovascular disease. In line with previous findings, the dementia subgroups had worse cognition and function, more severe medial temporal lobe atrophy, and elevated plasma neurofilament light chain, a marker of neurodegeneration; while VAD had significant neuroimaging measures of CeVD, and AD had elevated plasma pTau-181, a marker of amyloid pathology [17, 28]

**Table 1:** Baseline demographic and clinical characteristics of study participants

**Characteristics**	**NCI**	**CIND**	**AD**	**VaD**	***p-value***^a^
*Demographics*					
Maximum N^b^	93	217	166	50	
Age in Years, Mean (SD)	69.6 (7.0)	73.7 (7.8)	75.8 (7.3)	74.0 (8.3)	** < 0.001**
Female, N (%)	48 (51.6)	112 (51.6)	109 (65.7)	17 (34.0)	** < 0.001**
Above primary education, N (%)	64 (68.8)	116 (53.5)	50 (30.1)	17 (34.0)	** < 0.001**
*CeVD risk factors*					
Hypertension, N (%)^c^	55 (59.1)	148 (68.5)	118 (72.0)	50 (100.0)	** < 0.001**
Hyperlipidemia, N (%)^d^	64 (68.8)	168 (77.8)	115 (69.3)	45 (90.0)	**0.009**
Diabetes, N (%)	20 (21.5)	82 (37.8)	65 (39.2)	28 (56.0)	**0.001**
Cardiovascular disease, N (%)^e^	5 (5.4)	31 (14.3)	23 (13.9)	12 (25.0)	**0.014**
*Neuropsychological assessments*					
MMSE, median (IQR)^f^	28 (3)	24 (5)	16 (8)	15 (7)	** < 0.001**
MoCA, median (IQR)^g^	26 (3)	20 (6)	10 (8)	11 (6)	** < 0.001**
CDR-SOB, median (IQR)^h^	0 (0)	0.5 (1.0)	6.0 (5.0)	6.0 (7.0)	** < 0.001**
Global cognition z-score, median (IQR)^i^	0.13 (1.3)	-2.50 (2.7)	-6.62 (4.7)	-6.41 (5.7)	** < 0.001**
*Neuroimaging assessments*					
Significant CeVD, N (%)^j^	24 (26)	114 (53)	100 (62)	49 (100)	** < 0.001**
MTA score, median (IQR)^k^	1.0 (0)	1.0 (1.0)	2.0 (1.0)	2.0 (2.0)	** < 0.001**
*Plasma biomarker assessments*					
P-tau181, pg/mL, median (IQR)^l^	1.81 (1.0)	2.43 (1.8)	3.47 (2.7)	2.26 (1.9)	** < 0.001**
NfL, pg/mL, median (IQR)^m^	15.7 (8.7)	22.2 (13.4)	33.6 (26.5)	43.8 (51.1)	** < 0.001**

*Abbreviations*: *AD* Alzheimer’s disease, *CDR-SOB* Clinical Dementia Rating Scale Sum of Boxes, *CEVD* cerebrovascular diseases, *CIND* cognitively impaired, no dementia, *IQR* interquartile range, *MoCA* Montreal Cognitive Assessment, *MMSE* Mini-Mental State Examination, *MTA* medical temporal lobe atrophy, *N* number, *NCI* non-cognitively impaired, *NfL* neurofilament light chain, *P-tau181* tau phosphorylated at serine-181, *SD* standard deviation, *VaD* vascular dementia

^a^Values < 0.05 indicated significant group-wise differences using Kruskal–Wallis analyses of variance (age, neuropsychological assessment, MTA score, and plasma biomarkers) or Chi-square tests (sex, education, CeVD risk factors, presence of significance CeVD)

^b^Not all measurements were available for all participants due to a variety of reasons, including withdrawal of consent, inadequate samples or patient factors

^c^Measurement unavailable for 1 CIND, 2 AD

^d^Measurement unavailable for 1 CIND

^e^Measurement unavailable for 1 NCI, 2 VaD

^f^MMSE measured according to Folstein et al*.* [18]

^g^MoCA measured according to Nasreddine et al*.* [19]

^h^CDR-SOB measured according to O’Bryant et al. [29], measurement unavailable for 1 CIND

^i^Global cognition *z*-score derived from the neuropsychological battery (see Supplementary Table S2) by averaging the and standardizing the domain *z*-scores using the means and SDs of the NCI reference group as described previously [30], measurement unavailable for 2 AD

^j^Significant CeVD determined by presence of cortical infarcts, lacunes and / or white matter hyperintensities as previously described [16], measurement unavailable for 2 CIND, 4 AD and 1 VaD

^k^MTA scores measured according to Scheltens et al*.* [31], measurement unavailable for 2 AD, 2 VaD

^l^Plasma P-tau181 measurements as previously described [17], measurement unavailable for 10 NCI, 8 CIND, 13 AD and 6 VaD

^m^Plasma NfL measurements as previously described [28], measurement unavailable for 11 NCI, 8 CIND, 13 AD and 6 VaD

**Fig. 2:**
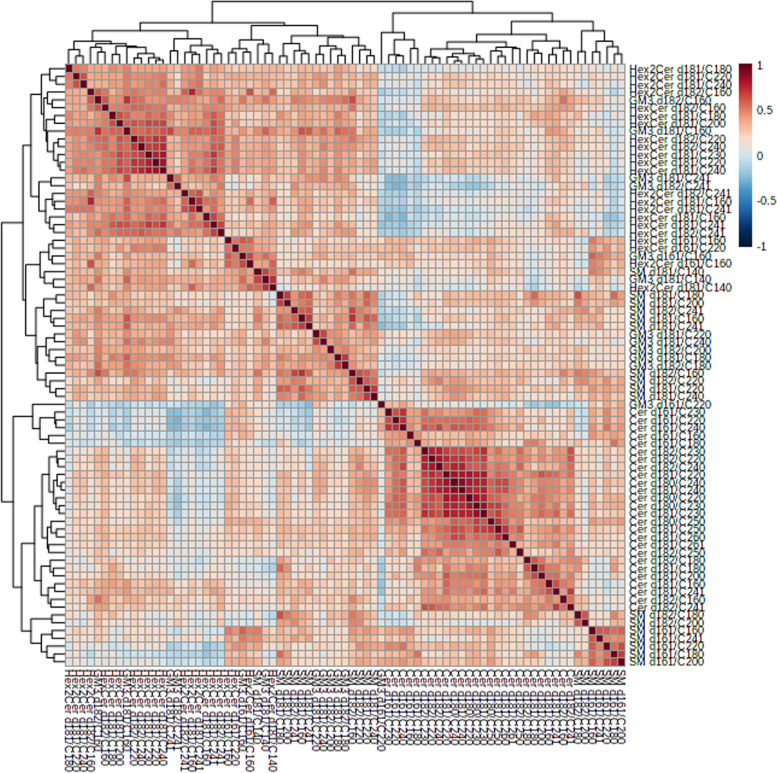
Heat-plot showing Spearman correlation coefficients of the sphingolipid species. Red represents positive correlations, blue negative. Clustering and heatmap conducted using Metaboanalyst 4.0

We obtained relative concentrations of 77 sphingolipid species (Supplementary Table 3) and evaluated the relationships among these species by Spearman’s correlation analysis. Generally, SM and Cer were found in separate clusters while HexCer and GM3 were clustered together. GM3 d16:1/C22:0 was also clustered with other Cer d16:1 s and SM d18:1/C14:0 with HexCer, Hex2Cer and GM3. d16:1 SPs were also found to be inversely correlated with d18:1 SPs (Fig. 2).

SPs concentrations were correlated with clinical characteristics (Supplementary Figure 2A-F, Supplementary Tables 4 and 5). In the presence of all the comorbidities explored, including hypertension, diabetes, cardiovascular disease and hyperlipidemia, SPs were generally decreased, especially HexCer, Hex2Cer and GM3 (Supplementary Figure 2A-E). The downregulation was also especially evident in the presence of cardiovascular disease (Supplementary Figure 2A, B). Interestingly, Cer were not significantly different between participants with and without diabetes (Supplementary Tables 4 and 5). In the presence of ApoE4 allele, no SPs were significantly altered although there was a trend towards increased SPs (Supplementary Figure 2A, F).

### Sphingolipid profiles differ in AD and VaD, compared to NCI

**Fig. 3:**
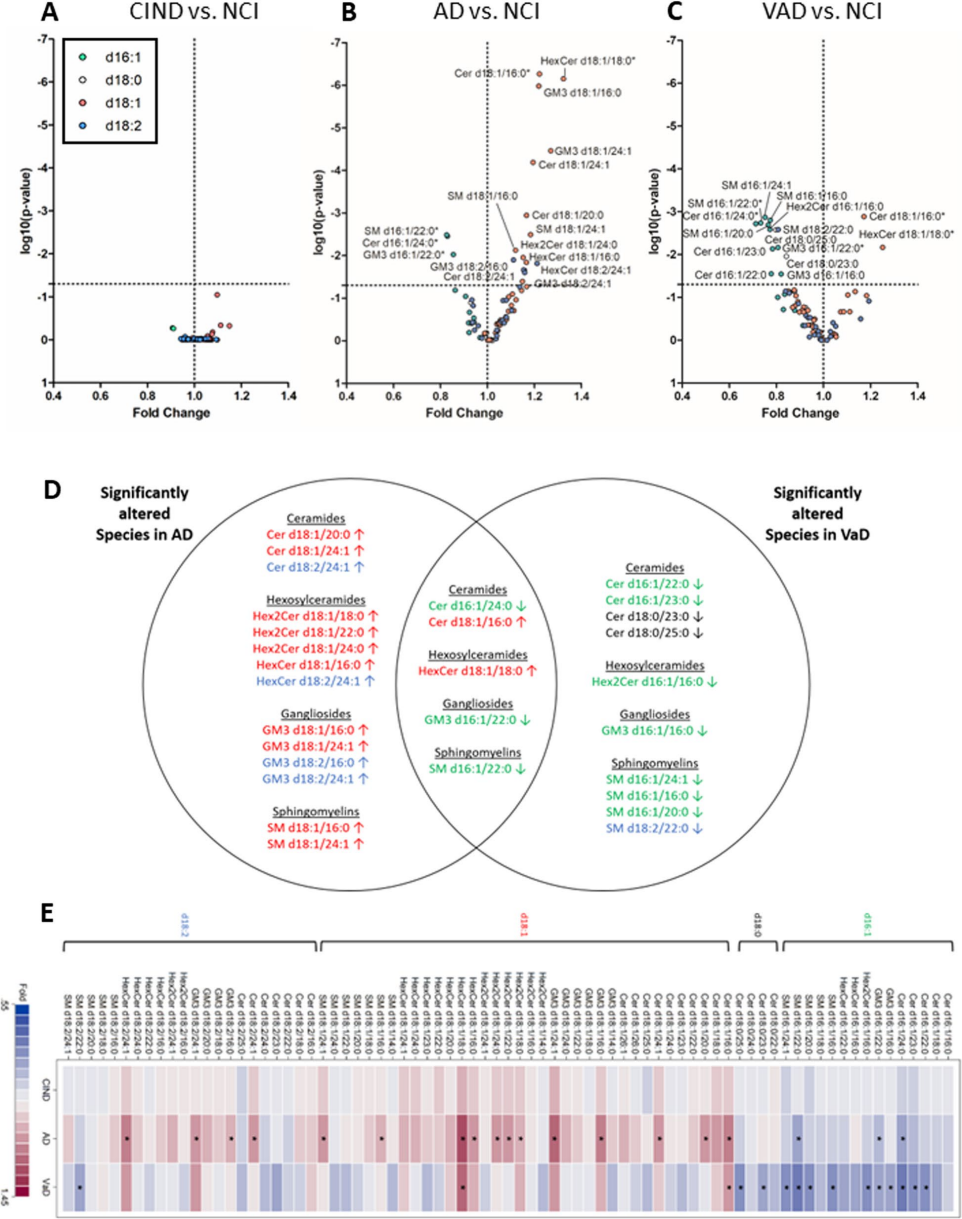
Volcano plots showing fold change of lipid concentration in **A** CIND, **B** AD and **C** VaD as compared to NCI versus significance of the relationship. Dotted horizontal line represents p-value = 0.05. Mann–Whitney U test was used and BH adjustment was conducted for p-values. Datapoints are coloured by sphingoid backbones. Green represents d16:1 backbone, red represents d18:1 backbone, blue represents d18:2 backbone, black represents d18:0 backbone. Species altered in both AD and VaD are labelled with*. **D** Venn diagram summarising the significantly altered species in AD and VaD, as compared to NCI. Lipids are coloured by sphingoid backbones. Green represents d16:1 backbone, red represents d18:1 backbone, blue represents d18:2 backbone, black represents d18:0 backbone. Arrows indicate if the species was increased (↑) or decreased (↓) as compared to NCI. **E** Heatplot showing the fold change of each lipid species in CIND, AD and VaD, as compared to NCI. Red indicates positive fold change, blue indicates negative fold change and shading intensity is proportional to level of fold change

We compared two subtypes of dementia in our study, AD and VaD. Profiles of SPs demonstrated that across all SP classes, species with d18:1 backbones were generally upregulated in AD, whereas d16:1 species were generally downregulated in VaD (Fig. 3B, C).

Out of 77 species, 5 SPs were altered in both dementias after false discovery rate correction, including upregulation of two d18:1 SPs (Cer d18:1/16:0 and HexCer d18:1/18:0) as well as downregulation of three d16:1 SPs (Cer d16:1/24:0, GM3 d16:1/22:0, SM d16:1/22:0). Fourteen other SPs were upregulated in AD, including 10 d18:1 SPs and 4 d18:2 SPs. On the other hand, 10 other SPs were downregulated in VaD, including seven d16:1 SPs, two d18:0 SPs and one d18:2 SP (Fig. 3B-E). No significant alterations were observed for CIND, relative to NCI, (Fig. 3A) but similar trends were observed where d18:1 SPs were increased and d16:1 SPs were decreased (Fig. 3D, Supplementary Tables 6 and 7).

Of the d18:1 SPs, those with n-acyl chains 16:0, 18:0, and 24:1 seems to be preferentially increased in both AD and VaD (Fig. 3E, Supplementary Figure 3A-B, Supplementary Table 7). This was observed across SP classes Cer, GM3, HexCer, and SM.

We also compared levels of plasma SPs in VaD, compared to AD, and found only 12 plasma SPs to be significantly decreased in VaD (Supplementary Figure 4). Given that plasma SPs are altered in the same direction in both subtypes of dementia but to differing extents, it is unsurprising that a direct comparison of plasma SPs between AD and VaD revealed small differences.

### Multiple sphingolipids were associated with risk of AD

**Fig. 4:**
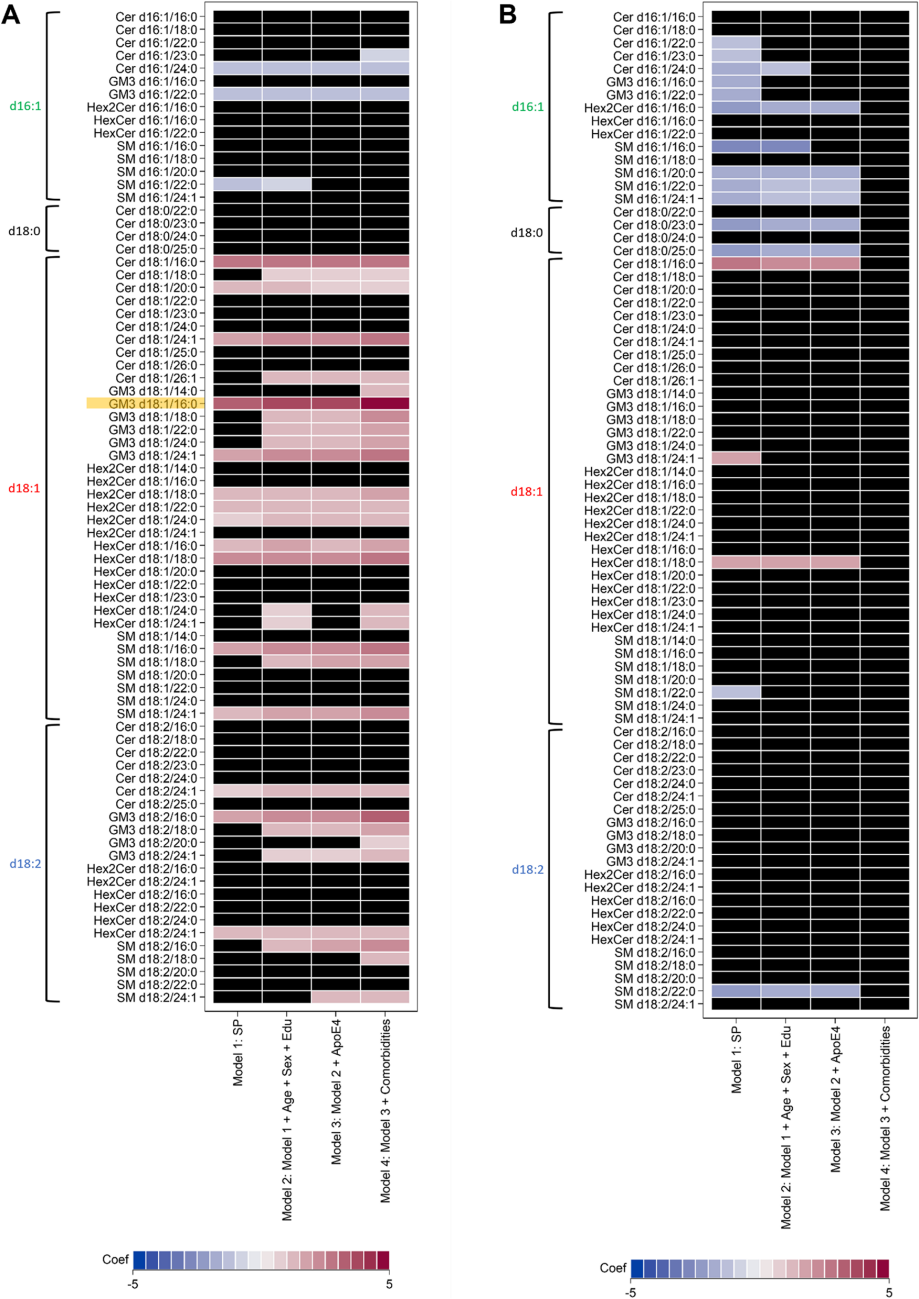
Logistic regression analyses between sphingolipid species (SP) and risk of **A** AD or **B** VaD. Models with increasing covariates are depicted from left to right. Shading intensity is proportional to coefficients. Relationships that are not statistically significant (BH-adjusted p-value > 0.05) are indicated with a black box. SP highlighted in yellow indicates the SP with highest odds ratio

To determine if SPs were significantly associated with risk of AD and VaD, we performed logistic regression analysis with increasing stringency (Fig. 4A-B, Supplementary Table 8). Individual SPs were used as predictor variables alone (Model 1), followed by the presence of demographic factors of age, sex and education level (Model 2), then with the addition of the ApoE4 allele (Model 3), and finally comorbidities including hypertension, hyperlipidemia, diabetes and cardiovascular disease (Model 4).

Eighteen SPs were significantly associated with risk of AD, mostly consisting of d18:1 SPs with positive associations with risk of AD. Four d16:1 SPs were found to be negatively associated. Interestingly, with increasing covariates in Model 2–4, both the number of associations and their statistical significance also increased (Fig. 4A, Supplementary Table 8). We investigated each comorbidity in separate models (Supplementary Figure 5A) and found that adjusting for the presence of diabetes dramatically increased the number of significant associations, including d18:2 SM, d18:2 Hex2Cer, and d18:1 HexCer. Diabetes has been reported to be tightly linked to dementia through insulin resistance, dyslipidemia as well as vascular events and, in particular, sphingolipids have been found to be a common factor in these processes [32]. We previously investigated these SPs in diabetes and reported them to be generally associated with decreased risk of diabetes [33]. Since diabetes is associated with increased risk of AD, the combination of these associations may have resulted in a masking effect when diabetes is not corrected for (i.e. increases in these SPs decrease risk of diabetes which in turn decrease risk of dementia, while simultaneously these SPs increase risk of dementia). For risk of VaD, seventeen SPs were significantly associated in Model 1, mostly consisting of d16:1 and d18:0 SPs with negative associations and d18:1 SPs with positive associations. Adjusting for covariates in Model 2–4 removed all significant associations (Fig. 4B, Supplementary Table 8). We investigated each comorbidity in separate models (Supplementary Figure 5B) and found that adjusting for presence of hypertension and cardiovascular disease removes most of the significant associations.

Interestingly, the majority of GM3s were found to be associated with risk of AD where, in particular, GM3 d18:1/16:0 and GM3 d18:1/24:1 were found with the strongest positive associations. On the other hand, these GM3 were not significantly associated with the risk of VaD (Fig. 4B, Supplementary Table 8).

### Plasma sphingolipids as diagnostic biomarkers for AD and VaD

We attempted to find the best subset of plasma SPs that can act as a multimarker panel for diagnosis of AD or VaD. We performed regression with Least Absolute Shrinkage and Selection Operator (LASSO) regularisation for variable selection [25] and utilised five-fold cross-validation to minimise predictive error and reduce overfitting [34]. LASSO regression was selected over the alternative stepwise regression, as stepwise regression may not give the best combination for our panel and it does not address overfitting.

**Fig. 5:**
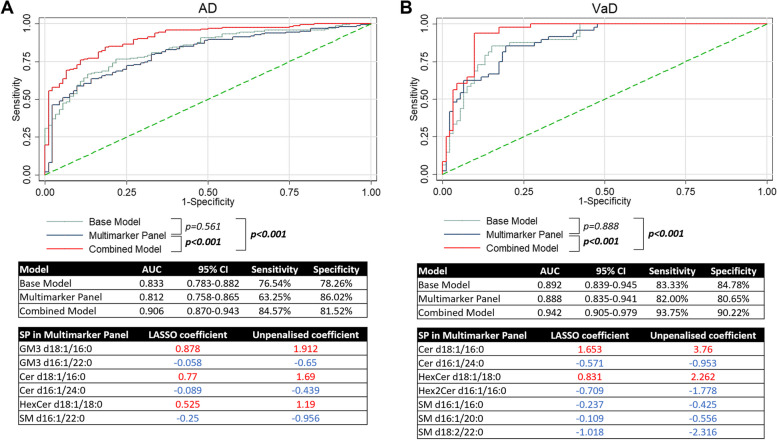
**A** ROC plot for base model of demographic factors and comorbidities, multimarker model with 6 SPs and combined model with SPs for AD. AUC with 95% confidence intervals, sensitivity and specificity of each model are reported. Table shows SPs selected by LASSO regression. Respective penalised and unpenalized coefficients are reported. **B** ROC plot for base model of demographic factors and comorbidities, multimarker model with 7 SPs and combined model with SPs for VaD. AUC with 95% confidence intervals, sensitivity and specificity of each model are reported. Table shows SPs selected by LASSO regression. Respective penalised and unpenalized coefficients are reported

LASSO regression resulted in a panel of six SPs for AD, including Cer d18:1/16:0, Cer d16:1/24:0, GM3 d18:1/16:0, GM3 d16:1/22:0, SM d16:1/22:0, and HexCer d18:1/18:0 (Fig. 5A). ROC analyses were performed to determine the diagnostic value of the panel, as well as the individual SPs selected, for discrimination of AD from NCI. The AUC for the panel was 0.812 while the AUC for individual SPs ranged from 0.620 to 0.713. Likelihood ratio tests were performed to compare each SP to the panel. Results showed that the panel performed better when compared to each SP (Supplementary Table 9).

The same set of analyses was performed for the discrimination of VaD from NCI. LASSO regression resulted in a panel of seven SPs for VaD, including Cer d18:1/16:0, Cer d16:1/24:0, SM d16:1/16:0, SM d16:1/20:0, SM d18:2/22:0, HexCer d18:1/18:0, Hex2Cer d16:1/16:0 (Fig. 5B). The AUC for the panel was 0.888 while the AUC for individual SPs ranged from 0.666 to 0.714. Similarly, the panel significantly improved the results when compared to each SP (Supplementary Table 10).

ROC analyses were performed and plotted for a base model of demographic factors and comorbidities, the multimarker panel as well as a combined base model with the multimarker panel for AD. Likelihood ratio test was performed to compare the base model to the combined model, as well as to compare the multimarker panel to the combined model. DeLong test was used to compare between base model and multimarker panel. The AUC for the base model was 0.833 (sensitivity = 76.54%, specificity = 78.26%). The AUC for the multimarker model was 0.812 (sensitivity = 63.25%, specificity = 86.02%). There was no significant difference in AUC between the base model and the multimarker model, although we observed that the base model has higher sensitivity whereas the SP multimarker panel has higher specificity. We compared these two models to the combined model with six SPs, which had a significantly improved AUC of 0.906 (sensitivity = 84.57%, specificity = 81.52%) (Fig. 5A). Addition of SPs to the base model increased both sensitivity and specificity.

Similar analyses were performed for VaD, the AUC for the base model was 0.892 (sensitivity = 83.33%, specificity = 84.78%). The AUC for the multimarker model was comparable at 0.888 (sensitivity = 82.00%, specificity = 80.65%). We compared these two models to the combined model with seven SPs which had a significantly improved AUC of 0.942 (sensitivity = 93.75%, specificity = 90.22%) (Fig. 5B). Again, we observed that addition of SPs to the base model increased both sensitivity and specificity.

**Fig. 6:**
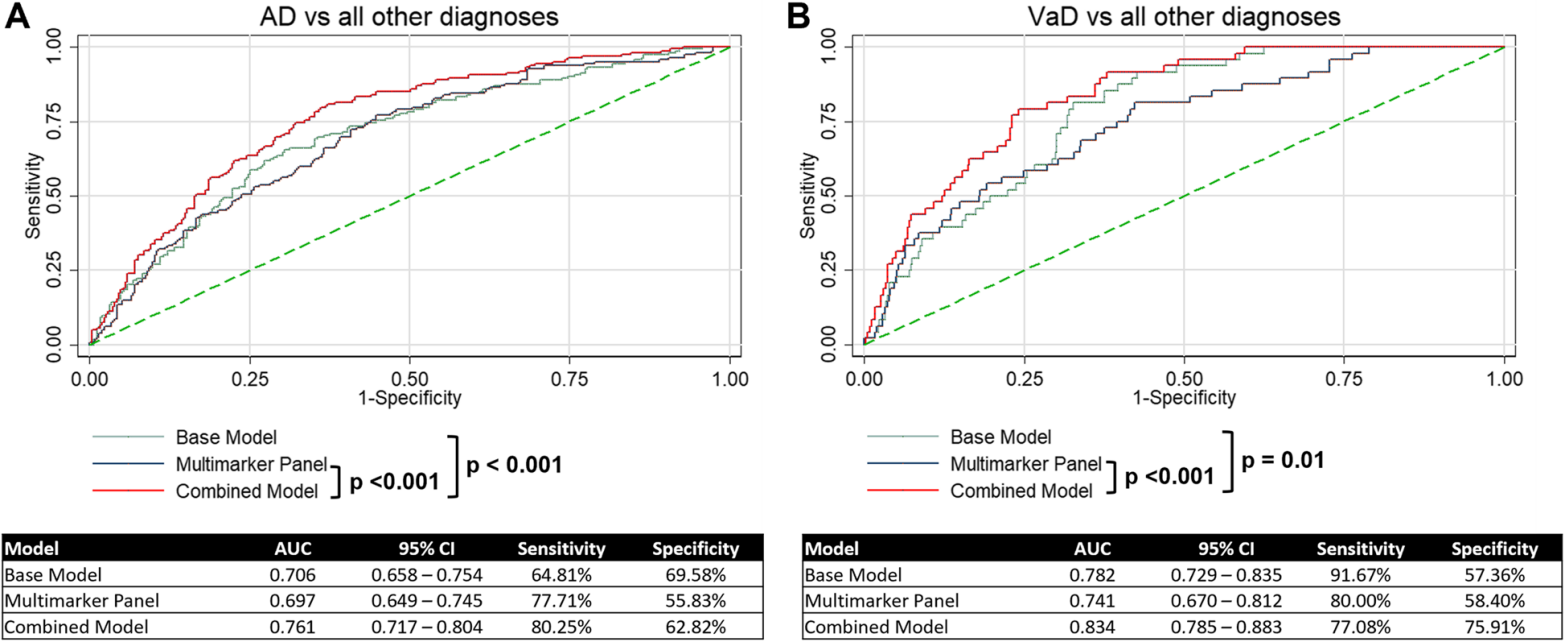
**A** ROC plot for baseline model of demographic factors and comorbidities, multimarker panel and combined model with SPs for AD to distinguish AD from all other diagnoses. **B** ROC plot for baseline model of demographic factors and comorbidities, and combined model with SPs for VaD to distinguish VaD from all other diagnoses. AUC with 95% confidence intervals, sensitivity and specificity of each model are reported

Backwards stepwise logistic regression was also performed, and a similar subset of SPs was selected for AD, except for the inclusion of HexCer d18:1/16:0 and exclusion of GM3 d16:1/22:0 as compared to the SPs selected by LASSO regression (Supplementary Table 11). Similarly for VaD, backwards stepwise logistic regression selected similar SPs except for the exclusion of SM d16:1/16:0 and SM d16:1/20:0 (Supplementary Table 12). Given that the population is not restricted to just AD and NCI, or VaD and NCI, it is sensible to determine the potential diagnostic utility of plasma SPs to discriminate AD or VaD from all other diagnoses (i.e. AD from NCI, CIND, VaD; or VaD from NCI, CIND, AD). ROC analyses were performed (Fig. 6A). The AUC for the base model was 0.706 for AD (sensitivity = 64.81%, specificity = 69.58%). The AUC for the multimarker model was comparable at 0.697 (sensitivity = 77.71%, specificity = 55.83%). Addition of 6 SPs to the base model significantly improved the AUC to 0.761 (sensitivity = 80.25%, specificity = 62.82%) (Fig. 6A). Similarly for VaD, the AUC for the base model was 0.782 (sensitivity = 91.67%, specificity = 57.36%). The AUC for the multimarker model was comparable at 0.741 (sensitivity = 80.00%, specificity = 58.40%). Addition of 7 SPs to the base model significantly improved the AUC to 0.834 (sensitivity = 77.08%, specificity = 75.91%) (Fig. 6B).

## Discussion

Previous sphingolipidomic studies typically quantified SP classes in aggregate or looked at only the d18:1 SPs [35–38]. While these studies demonstrate that AD is associated with significant alterations in SP accumulation, the depth of their reported SP profiles were incomplete, limiting the scope of the observations and likely contributing to variations in study conclusions. Apart from the canonical d18:1 sphingoid backbone, humans synthesise and utilise sphingolipids containing other sphingoid backbones, such as d16:1 [39, 40]. In this study, we examined sphingoid backbone-specific changes in SPs of AD and VaD patients and observed multiple d18:1 sphingoid backbone-specific elevations in AD. We also report for the first time plasma SPs alterations in VaD.

### AD and VaD subjects have varying alterations to the patterns of their sphingolipid profiles

Our results demonstrate that d18:1 SP content is higher and d16:1 SP content is lower in the plasma of dementia patients, as compared to healthy controls. The contrast in d18:1 and d16:1 SPs alterations across all classes may also explain the inconsistent results observed in past studies that often quantified SPs by their classes. This highlights the critical importance of capturing species-specific changes.

Apart from the opposing direction of change between d18:1 and d16:1 SPs in dementia, we found an interesting contrast between AD and VaD. The majority of altered SPs in AD were upregulated d18:1 SPs while the majority in VaD were downregulated d16:1 SPs. A recent meta-analysis of two large-scale studies in Australia also analysed species-specific changes in the lipidome of AD patients. This study reported no apparent differences between sphingoid bases [41]. Our results demonstrated otherwise, this could be likely due to the fact that the Australian study only included AD patients while in our study, we found that the contrast between the sphingoid bases was made more evident as we compared the sphingolipid profile of AD against VaD, and the downregulation of d16:1 content was more prominent and apparent in VaD.

Studies and reviews have emphasised that AD and VaD have overlapping pathophysiologies and pathogenic factors that may act in additive or synergistic ways. For example, characteristics of AD such as amyloid plaques are found in VaD patients, and conversely, cerebrovascular diseases such as microbleeds and infarcts are found in AD patients. Both share common pathogenic factors, such as inflammation, vascular changes and apoptosis [42]. Nonetheless, these two dementia subtypes still vary in terms of prognosis, and their pathophysiologies may have distinct underlying mechanisms. This is highlighted in our study, where we found distinct changes in SP profiles between AD and VaD.

Global downregulation of SPs, regardless of SP class or sphingoid backbone, was associated with all systemic vascular diseases explored in our study, including hypertension, diabetes, and cardiovascular disease. These diseases are risk factors for dementia and have been found to be associated with risk of vascular events such as myocardial infarction and stroke, leading to cerebrovascular diseases [10, 43]. VaD is tightly linked to such vascular events. Moreover, our results demonstrate that, after adjustment for these vascular risk factors, none of the SPs remained significantly associated with risk of VaD. We postulate that the downregulation of SPs in VaD is mainly contributed by these vascular risk factors for VaD. Future studies are required to determine whether derangement of SPs is a cause or a consequence of cardiovascular disease. However, it is worth noting that we previously identified genetic determinants of d16:1 SP content [44], suggesting that such changes may precede cardiovascular disease. Our results add to the expanding literature that SPs may be intricately linked to other important vascular diseases apart from cerebrovascular disease.

In contrast to the d16:1-specific reductions in VaD, we observed specific increases in d18 SPs in AD patients that remained significant after adjustment for these vascular factors. This highlights the possibility that the increase in d18 SPs is driven directly by AD-specific pathology, or alternatively, that increased levels of d18:1 SPs represent a novel AD risk factor.

### Sphingoid backbone-specific alterations in dementia

The alterations observed were not specific to a particular class of SPs, rather the changes were seen across all classes of SPs investigated. This suggests that the changes we observed may be the result of alterations to de novo SP synthesis. Sphingoid backbone chain length is determined during the first step of de novo synthesis by serine palmitoyltransferase (SPT). This heterotrimeric enzyme complex consists of SPT Long Chain Base Subunit 1 (SPTLC1), either SPTLC2 or SPTLC3, and either SPT Small Subunit a (SPTssa) or SPTssb. The combination of the subunits influences substrate preference of SPT, in particular incorporation of SPTLC2 preferentially generates the d18:1 backbone from a palmitoyl-CoA substrate, whereas SPTLC3 preferentially generates the d16:1 backbone from myristoyl-CoA [45]. One study investigating SPTLC protein expression in post-mortem brain tissues found SPTLC2 to be elevated in AD brain samples [46], consistent with an increase in d18:1 SPs. To our knowledge, no studies have evaluated the role of SPTLC3 in cognitive impairment. In other cardiovascular disease models, a genome-wide association study (GWAS) has reported SPTLC3 genetic variants to be associated with protective odds for myocardial infarction [47]. Since associations between cardiovascular events and dementia have often been cited [48], it is plausible that SPTLC3 or other SP synthesis-associated enzymes may be playing important roles in pathophysiology of dementia. Further research is required to explore this.

### Are d18:1 and d16:1 sphingolipids functionally different?

In this study, we show that SPs with structurally distinct sphingoid backbones vary in the directions in which they are altered in dementia, and that they differ to varying extents among dementia subtypes. These findings raise the question of whether sphingolipids differ functionally based on their sphingoid backbones. Limited studies have addressed this question as detailed investigations into sphingoid backbones were only made possible by recent advances in lipidomics.

Our group recently identified functional differences between d16:1 and d18:1 sphingosine 1-phosphates (S1P) in inflammatory signalling whereby d16:1 attenuates d18:1 pro-inflammatory signalling [49]. Other groups have shown similar work in cardiomyocytes, whereby d18 dihydrosphingosine (DHS) was shown to induce autophagy while d16 DHS was shown to inhibit cell viability [50]. These studies illustrate potential differences in functions of signalling SPs with different sphingoid backbone. Given that the SPs explored in our current study are not only involved in signalling but also in influencing membrane properties [51], varying sphingoid backbone may exert a differential effect on these processes in dementia.

### Involvement of gangliosides in AD

Interestingly, we found GM3 d18:1/16:0 and GM3 d18:1/24:1 to be the most positively associated with risk of AD. This is corroborated by the recent meta-analysis which also identified GM3 d18:1/24:1 to have the strongest positive association with risk of AD [41]. In post-mortem studies of AD, GM3 has been found to be elevated in AD, and in subcortical ischemic vascular dementia and mixed dementia, upregulation of GM3 d18:1/16:0 and GM3 d18:1/24:1 have been observed [12, 52].

Gangliosides are sialylated glycosphingolipids that are localised mostly to the plasma membrane in neuronal lipid rafts. They are known to play roles in various processes such as receptor interactions and apoptosis. GM3 is one of the simpler gangliosides that predominate in the periphery while it is found in lower levels in the central nervous system. Shifts from complex to simple gangliosides such as GM3 have been reported in aging brains as well as brains with AD [53]. As a precursor to the array of complex gangliosides, GM3 can be converted by GM2-synthase to form a-series gangliosides such as GM2 and GM1. Knockout of GM2-synthase in AD mice model reportedly led to the accumulation of GM3 and increased amyloid burden [54, 55]. GM3 can also be converted by GD3-synthase into b-series gangliosides such as GD3 which have been found to be involved in amyloid production. Moreover, knockout of GD3-synthase in AD mice model showed reduction in AD pathology [54, 56]. Another study evaluated the effect of GM3-synthase knockout as well as treatment with sialic acid binding lectin to reduce all gangliosides in AD mice. This resulted in reduced amyloid burden and decreased inflammation, as well as increased synaptic markers and improved cognitive function [57]. These studies point to the potential role GM3 may play in amyloid pathology and the pathogenesis of AD. Although our study did not quantify the complex gangliosides, we found a strong upregulation of GM3 in AD patients and add on to the literature that GM3 may be a plausible therapeutic target for AD.

### Multimarker models are more sensitive and specific for classifying AD or VaD from NCI as well as all other diagnoses

Our results also highlighted the utility of a multimarker panel in delineating dementia from NCI patients as well as all other diagnoses. The analyses to distinguish AD or VaD from all other diagnoses is more representative of the population, as compared to distinguishing AD or VaD from NCI alone. For the ROC analyses to distinguish AD or VaD from all other diagnoses, we found that utilising plasma SPs multimarker panels alone (AUC = 0.697 for AD, AUC = 0.741 for VaD) can reasonably discriminate AD or VaD from all other diagnoses. Furthermore, when we compared a base model of demographic factors and comorbidities known to be associated with risk of dementia to a base model combined with plasma SPs, we found the AUC to be significantly improved for diagnoses of AD (AUC = 0.761) and VaD (AUC = 0.834). The inclusion of multiple SPs may have clinical utility as diagnostic markers for AD and VaD in the population. Furthermore, we utilised LASSO regression with cross-validation to minimise out-of-sample prediction error to find the best combination of SPs for the multimarker panel [25]. λ_1SE_ was also chosen to find the simplest model with comparable accuracy to the best model [26]. This is ideal for biomarkers selection to minimise time for processing while maintaining accuracy of the panel.

While LASSO regression is superior to alternative methods such as stepwise regression to derive the best model, it comes with its own set of limitations. For example, if there are more than 1 collinear variable, LASSO regression arbitrarily drops 1. Hence, the final selected covariates are known to belong to the true model or are correlated to those that belong, whereas covariates omitted by LASSO regression do not belong to the true model or belongs but are correlated to those that are already found within the model. Nonetheless, we utilised LASSO regression to find the best multimarker panel of SPs for diagnostic purposes to discriminate AD or VaD from NCI and not for the purpose of inference. This is also with reference to other studies that have also utilised LASSO regression to determine the best subset of lipids for diagnostic purposes [11].

## Conclusions

Our study identified specific changes to SPs in the human plasma sphingolipidome and found that, 1) d16:1 and d18:1 SPs were regulated differently in dementia and, 2) SP backbones are altered to varying extent depending on the subtype of dementia. Previous studies have emphasised the complexity of sphingolipid signalling and the value in studying alterations in all species. Our study further provided an additional perspective that the sphingoid backbones of SPs need to be evaluated and considered as a factor for further investigation in sphingolipid research. While the line between AD and VaD is often blurred and it is difficult to delineate these two subtypes of dementia, our study underscores a meaningful difference between these two dementias with the apparent difference in SPs regulation based on their sphingoid backbone structures. We also found that gangliosides alterations may be associated with AD diagnosis.

While we provided an overview of the changes in plasma SPs in dementia, each SP class is involved in multiple processes that may be associated with the disease. We are unable to conclude whether changes in SPs are causative or a sequela of the disease, nor can we delineate the disease processes in which the altered SPs are involved. Hence, more mechanistic studies are required to determine and investigate this. In addition, SPs are highly diverse and although we captured the major classes of SPs, we did not quantify some of the other classes such as complex gangliosides, sphingosines and ceramide 1-phosphates which may also be involved in the pathophysiology of dementia. Furthermore, our suggested multimarker panels as diagnostic markers for AD and VaD will still require a validation cohort to test for external validity as well as clinical utility. Nonetheless, we present a novel difference between the two most common subtypes of dementias and bring attention to the importance of capturing species-specific changes.

### Supplementary Information

**Additional file 1: Supplementary Table S1.** Diagnostic criteria of clinical subgroups for this study. **Supplementary Table S2.** Summary of Neuropsychological Battery and Component Tests. **Supplementary Table S3.** List of quantifiable lipid species in this study. **Supplementary Table S4.** Median concentrations of SPs in absence or presence of comorbidity or ApoE4 allele. **Supplementary Table S5.** SPs fold change and BH-*p* values in presence versus absence of comorbidity or ApoE4 allele. **Supplementary Table S6.** Median concentrations (nM) of SPs in NCI, CIND, AD and VaD. **Supplementary Table S7.** SPs Fold change and BH-*p* values in CIND, AD, VaD compared to NCI. **Supplementary Table S8.** Associations of SPs and risk of AD or VaD diagnoses, expressed as odds ratio (OR) and BH-adjusted *p*-values. **Supplementary Table S9.** AUC values for individual SPs and selected multimarker panel for AD. **Supplementary Table S10.** AUC values for individual SPs and selected multimarker panel for VaD. **Supplementary Table S11.** Variables selected by stepwise backwards regression for AD. **Supplementary Table S12.** Variables selected by stepwise backwards regression for VaD.

**Additional file 2: Supplementary Figure S1.** PCA analysis plots for complete lipidomic run, demonstrating no batch effects. Green represents samples, red represents pooled quality control (PQC) and blue represents technical quality control (TQC). **Supplementary Figure S2.** **A** Heatplot showing the fold change of each sphingolipid species in presence vs. absence of comorbidity or ApoE4 allele. **B-F** Volcano plots showing fold change of lipid concentration in presence of comorbidity or ApoE4 allele as compared to absence versus significance of the relationship. Dotted horizontal line represents *p*-value = 0.05. Mann-Whitney U test was used and BH adjustment was conducted for *p*-values. Datapoints are coloured by sphingoid backbones. Green represents d16:1 backbone, red represents d18:1 backbone, blue represents d18:2 backbone, black represents d18:0 backbone. Scale for y-axis was reduced to better illustrate the spread of the datapoints in **F**. **Supplementary Figure S3.** Volcano plots showing fold change of lipid concentration in **A** AD and **B** VaD as compared to NCI versus significance of the relationship. Dotted horizontal line represents *p*-value = 0.05. Mann-Whitney U test was used and BH adjustment was conducted for *p*-values. Datapoints are coloured by n-acyl chain lengths. Pink represents short chain acyls (C14), red represents long chain acyls (C16-18), green represents very long chain acyls (C20-26), blue represents monounsaturated acyls (C24:1). **Supplementary Figure S4.** **A** Volcano plots showing fold change of lipid concentration in VaD as compared to AD versus significance of the relationship. Dotted horizontal line represents *p*-value = 0.05. Mann-Whitney U test was used and BH adjustment was conducted for *p*-values. Datapoints are coloured by sphingoid backbones. Green represents d16:1 backbone, red represents d18:1 backbone, blue represents d18:2 backbone, black represents d18:0 backbone. **B** List of species significantly different between AD and VaD, with fold change and p-value stated. **Supplementary Figure S5.** Logistic regression analyses between sphingolipid species (SP) and risk of **A** AD or **B** VaD, adjusted for separate comorbidities. Models with increasing covariates are depicted from left to right. Shading intensity is proportional to coefficients. Relationships that are not statistically significant (BH-adjusted p-value > 0.05) are indicated with a black box.

**Additional file 3. **Supplementary Information: Covariates.

## Data Availability

The data presented in this study are available in the manuscript and supplementary materials.

## Abbreviations

AD: Alzheimer's Disease
ANOVA: Analyses of Variance
APOE: Apolipoprotein E
AUC: Area Under Curve
Cer: Ceramide
CIND: Cognitive Impairment No Dementia
CoV: Coefficient of Variation
DHS: Dihydrosphingosine
DSM-IV: Diagnostic and Statistical Manual Fourth Edition
FDR: False Discovery Rate
GM: Ganglioside
GWAS: Genome-Wide Association Study
Hex2Cer: Dihexosylceramide
HexCer: Monohexosylceramide
LASSO: Least Absolute Shrinkage and Selection Operator
NCI: No Cognitive Impairment
NINCDS-ADRDA: National Institute of Neurological and Communicative Disorders and Stroke and the Alzheimer’s disease and Related Disorders Association
NINDS-AIREN: National Institute of Neurological Disorders and Stroke-Association Internationale pour la Recherché et l’ Enseignement en Neuroscience
QC: Quality Control
ROC: Receiver-Cperating Characteristics
S1P: Sphingosine 1-Phosphate
SM: Sphingomyelin
SP: Sphingolipid
SPT: Serine Palmitoyltransferase
SPTLC1: Serine Palmitoyltransferase Long Chain Base Subunit 1
SPTLC2: Serine Palmitoyltransferase Long Chain Base Subunit 2
SPTLC3: Serine Palmitoyltransferase Long Chain Base Subunit 3
SPTssa: Serine Palmitoyltransferase Small Subunit a
SPTssb: Serine Palmitoyltransferase Small Subunit b
VaD: Vascular Dementia
