# Supplementary material for: Lipidomics profiling reveals distinct patterns of plasma sphingolipid alterations in Alzheimer’s disease and vascular dementia
Source: Alzheimers Res Ther. 2023 Dec 12;15:214. doi: 10.1186/s13195-023-01359-7 (PMC10714620; doi:10.1186/s13195-023-01359-7)
Supplement: Supplementary file 2 — Additional file 2: Supplementary Figure S1. PCA analysis plots for complete lipidomic run, demonstrating no batch effects. Green represents samples, red represents pooled quality control (PQC) and blue represents technical quality control (TQC). Supplementary Figure S2. A Heatplot showing the fold change of each sphingolipid species in presence vs. absence of comorbidity or ApoE4 allele. B-F Volcano plots showing fold change of lipid concentration in presence of comorbidity or ApoE4 allele as compared to absence versus significance of the relationship. Dotted horizontal line represents p-value = 0.05. Mann-Whitney U test was used and BH adjustment was conducted for p-values. Datapoints are coloured by sphingoid backbones. Green represents d16:1 backbone, red represents d18:1 backbone, blue represents d18:2 backbone, black represents d18:0 backbone. Scale for y-axis was reduced to better illustrate the spread of the datapoints in F. Supplementary Figure S3. Volcano plots showing fold change of lipid concentration in A AD and B VaD as compared to NCI versus significance of the relationship. Dotted horizontal line represents p-value = 0.05. Mann-Whitney U test was used and BH adjustment was conducted for p-values. Datapoints are coloured by n-acyl chain lengths. Pink represents short chain acyls (C14), red represents long chain acyls (C16-18), green represents very long chain acyls (C20-26), blue represents monounsaturated acyls (C24:1). Supplementary Figure S4. A Volcano plots showing fold change of lipid concentration in VaD as compared to AD versus significance of the relationship. Dotted horizontal line represents p-value = 0.05. Mann-Whitney U test was used and BH adjustment was conducted for p-values. Datapoints are coloured by sphingoid backbones. Green represents d16:1 backbone, red represents d18:1 backbone, blue represents d18:2 backbone, black represents d18:0 backbone. B List of species significantly different between AD and VaD, with fold change and p-valu [file 13195_2023_1359_MOESM2_ESM.pdf]

Chua *et al.* Lipidomics profiling reveals distinct patterns of plasma sphingolipid alterations in Alzheimer's disease and vascular dementia

Additional file 2

Supplementary Figure S1

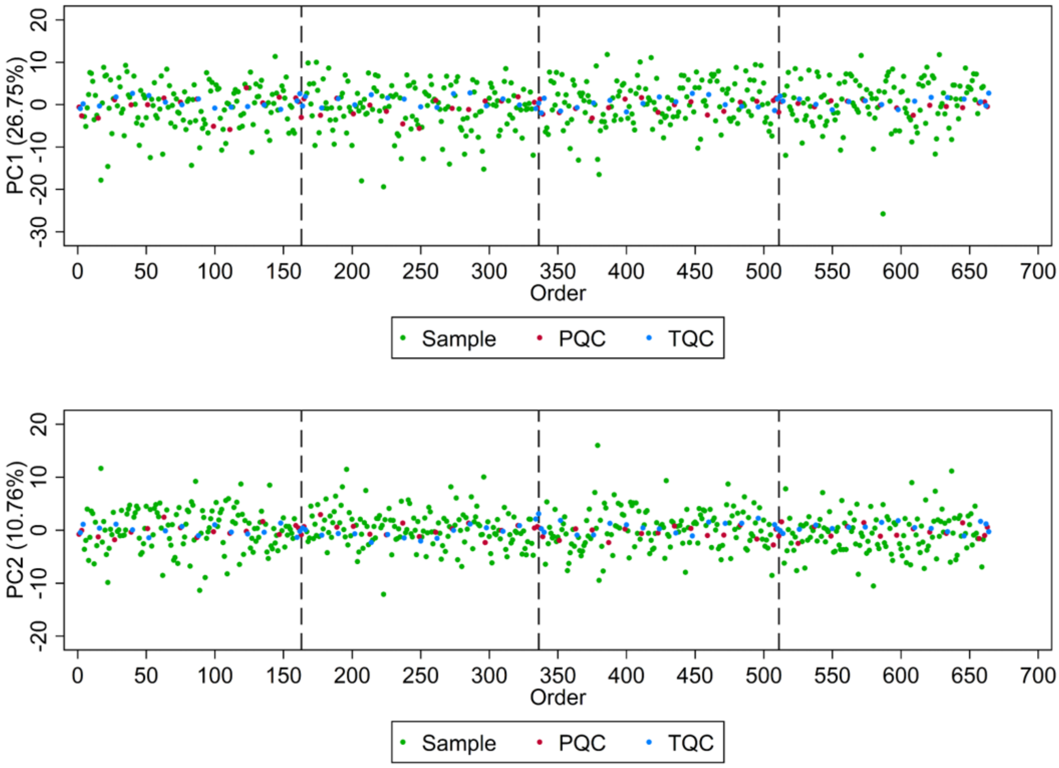

PCA analysis plots for complete lipidomic run, demonstrating no batch effects. **Green** represents samples, **red** represents pooled quality control (PQC) and **blue** represents technical quality control (TQC).

Supplementary Figure S2

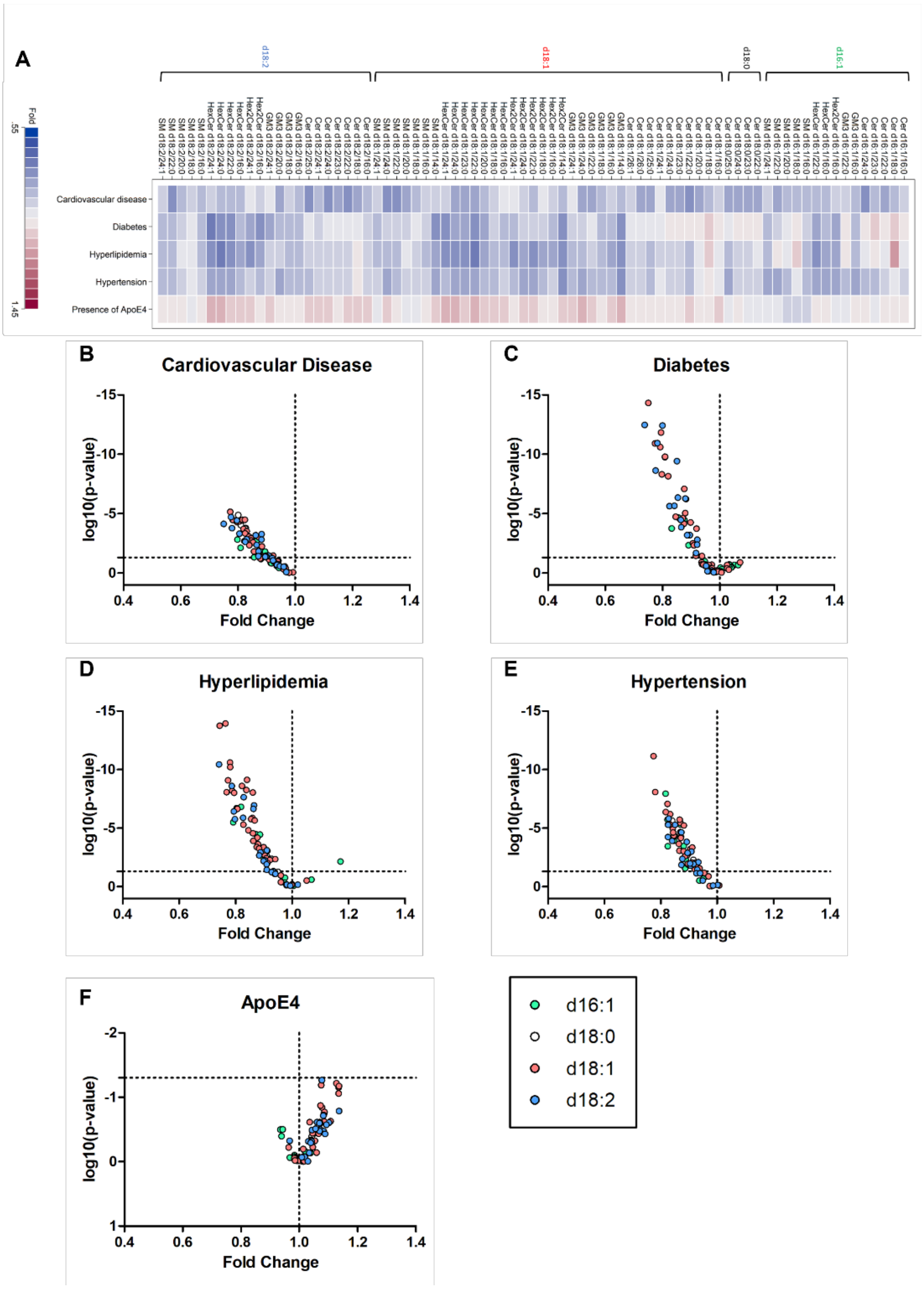

A Heatplot showing the fold change of each sphingolipid species in presence vs. absence of comorbidity or ApoE4 allele. **B-F** Volcano plots showing fold change of lipid concentration in presence of comorbidity or ApoE4 allele as compared to absence versus significance of the relationship. Dotted horizontal line represents p-value = 0.05. Mann-Whitney U test was used and BH adjustment was conducted for p-values. Datapoints are coloured by sphingoid backbones. Green represents d16:1 backbone, red represents d18:1 backbone, blue represents d18:2 backbone, black represents d18:0 backbone. Scale for y-axis was reduced to better illustrate the spread of the datapoints in **F**.

Supplementary Figure S3

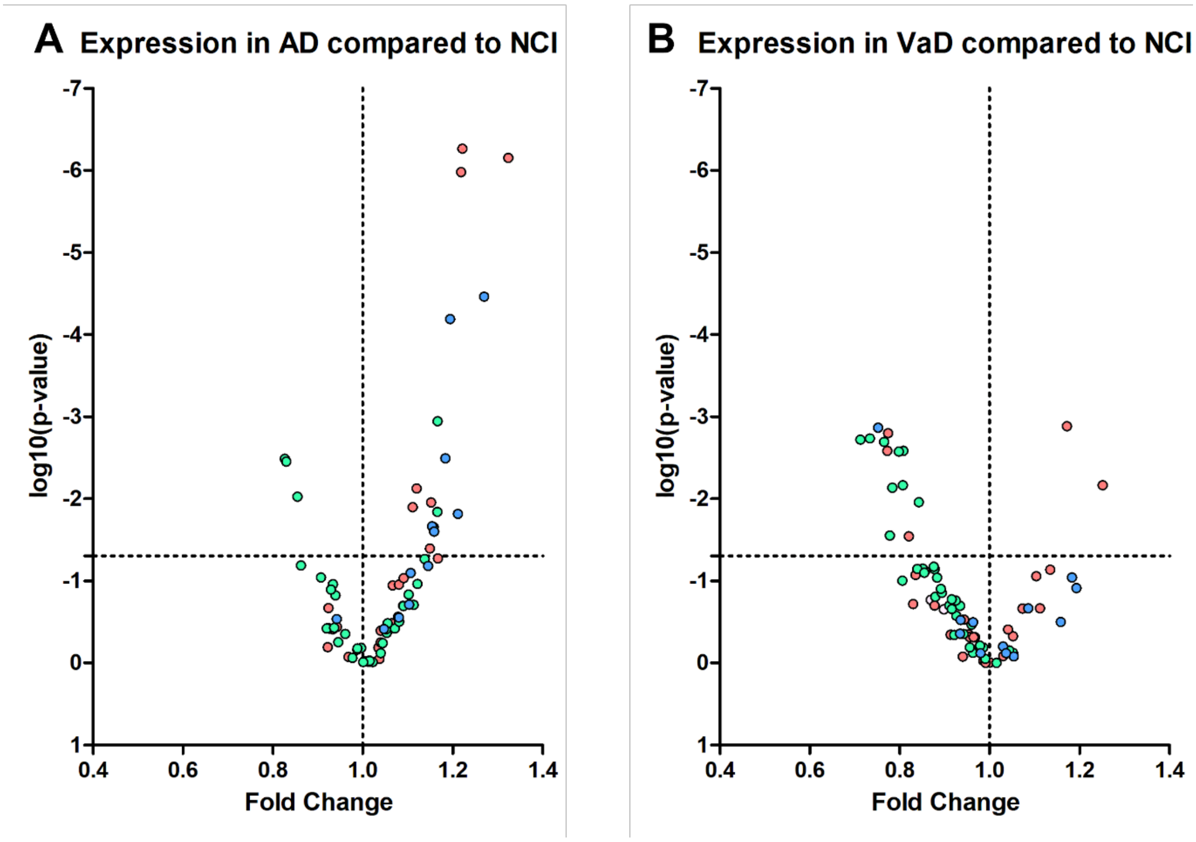

Volcano plots showing fold change of lipid concentration in **A** AD and **B** VaD as compared to NCI versus significance of the relationship. Dotted horizontal line represents p-value = 0.05. Mann-Whitney U test was used and BH adjustment was conducted for p-values. Datapoints are coloured by n-acyl chain lengths. **Pink** represents short chain acyls (C14), **red** represents long chain acyls (C16-18), **green** represents very long chain acyls (C20-26), **blue** represents monounsaturated acyls (C24:1).

Supplementary Figure S4

A

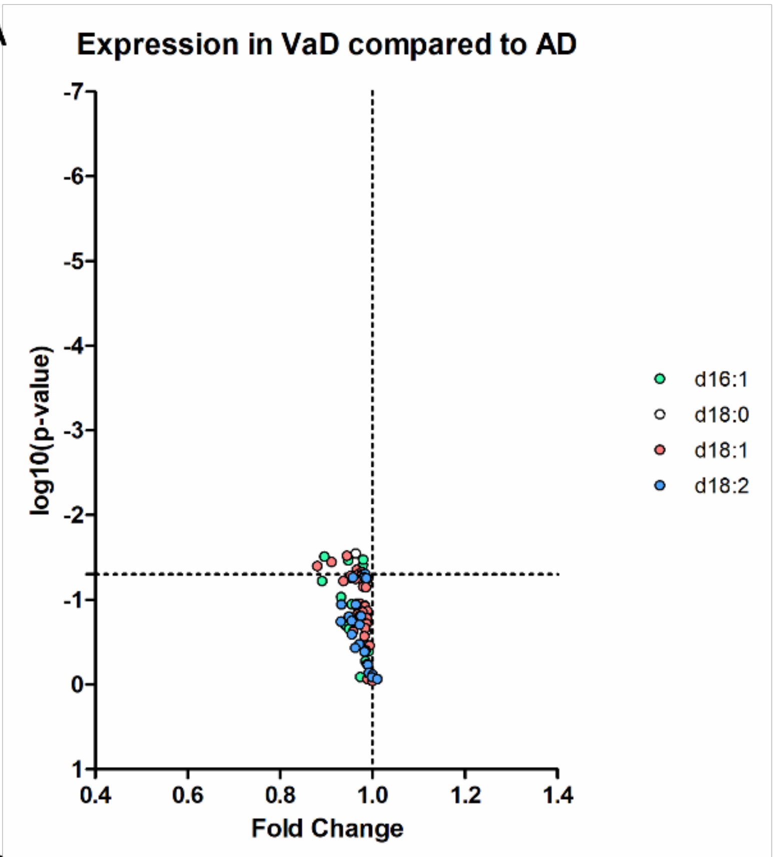

B

Plasma SP	Fold change	P-value
Cer d18:0/C23:0	0.963	0.028
Cer d18:1/C26:0	0.911	0.036
GM3 d16:1/C16:0	0.896	0.031
GM3 d18:1/C14:0	0.88	0.04
Hex2Cer d16:1/C16:0	0.948	0.034
Hex2Cer d18:1/C22:0	0.945	0.03
HexCer d18:1/C22:0	0.969	0.05
HexCer d18:1/C23:0	0.966	0.044
HexCer d18:1/C24:0	0.977	0.047
SM d16:1/C16:0	0.979	0.039
SM d16:1/C20:0	0.98	0.034
SM d18:2/C24:1	0.984	0.049

A Volcano plots showing fold change of lipid concentration in VaD as compared to AD versus significance of the relationship. Dotted horizontal line represents p-value = 0.05. Mann-Whitney U test was used and BH adjustment was conducted for p-values. Datapoints are coloured by sphingoid backbones. Green represents d16:1 backbone, red represents d18:1 backbone, blue represents d18:2 backbone, black represents d18:0 backbone. **B** List of species significantly different between AD and VaD, with fold change and p-value stated.

Supplementary Figure S5

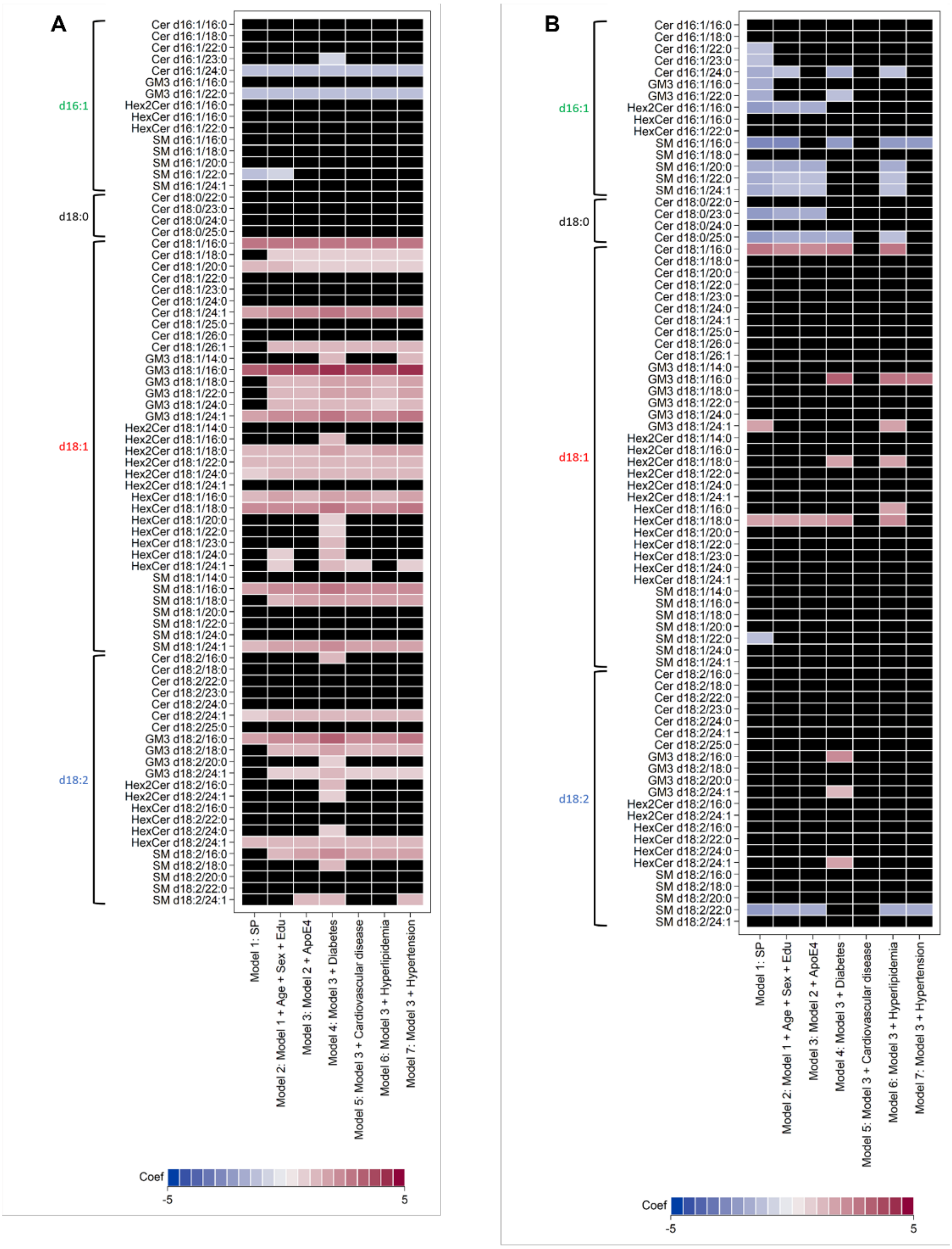

Logistic regression analyses between sphingolipid species (SP) and risk of **A** AD or **B** VaD, adjusted for separate comorbidities. Models with increasing covariates are depicted from left to right. Shading intensity is proportional to coefficients. Relationships that are not statistically significant (BH-adjusted p-value > 0.05) are indicated with a **black** box.
