# Supplementary material for: Lipidomics profiling reveals distinct patterns of plasma sphingolipid alterations in Alzheimer’s disease and vascular dementia
Source: Alzheimers Res Ther. 2023 Dec 12;15:214. doi: 10.1186/s13195-023-01359-7 (PMC10714620; doi:10.1186/s13195-023-01359-7)
Supplement: Supplementary file 3 — Additional file 3. Supplementary Information: Covariates. [file 13195_2023_1359_MOESM3_ESM.pdf]

Chua *et al.* Lipidomics profiling reveals distinct patterns of plasma sphingolipid alterations in Alzheimer's disease and vascular dementia

Additional file 3.

Supplementary Information: Covariates

Vascular risk factors such as hypertension, hyperlipidemia, diabetes, smoking and cardiovascular disease were collected and classified as absent or present. Hypertension was defined as systolic blood pressure ≥ 140mmHg and /or diastolic blood pressure ≥ 90mmHg or a history of hypertension, or use of antihypertensive medication. Hyperlipidaemia was defined as total cholesterol level ≥ 4.14 mmol/l or a history of hyperlipidemia, or use of lipid-lowering medication. Diabetes mellitus was defined as glycated hemoglobin ≥ 6.5% or a history of diabetes mellitus, or the use of any glucose-lowering medication. Cardiovascular disease was determined by previous history of atrial fibrillation, congestive heart failure and / or myocardial infarction. Apolipoprotein E (APOE) genotyping were as previously described[1] for the determination of APOE ε4 carrier status, defined by the presence of at least one APOE ε4 allele.

REFERENCES

1. Chai YL, Yeo HK-H, Wang J, *et al*. Apolipoprotein ε4 is associated with dementia and cognitive impairment predominantly due to Alzheimer's disease and not with vascular cognitive impairment: a Singapore-based cohort. *Journal of Alzheimer's Disease*. 2016;51(4):1111-1118.
